# Supplementary material for: Splice-Junction-Based Mapping of Alternative Isoforms in the Human Proteome
Source: Cell Rep. Author manuscript; Available in PMC 2020 Jan 15. (PMC6961840; doi:10.1016/j.celrep.2019.11.026)
Supplement: 3 [file NIHMS1546469-supplement-3.zip › DF2/PXD009737/TestisGluC-143-Q9NZM3-MDSFNNQLKE.pdf]

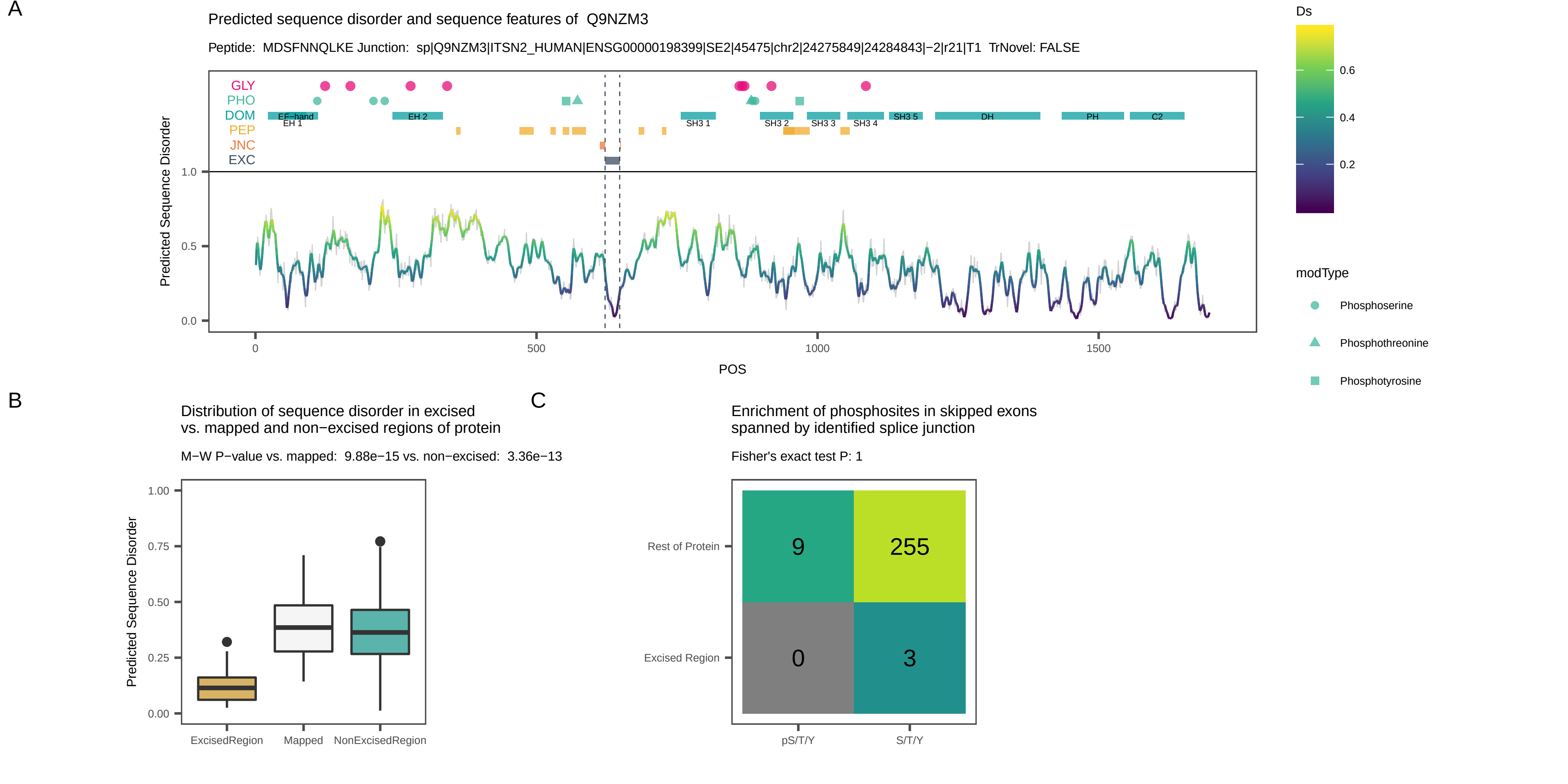

A
Predicted sequence disorder and sequence features of Q9NZM3
Peptide: MDSFNNQLKE Junction: sp|Q9NZM3|ITSN2_HUMAN|ENSG00000198399|SE2|45475|chr2|24275849|24284843|−2|r21|T1 TrNovel: FALSE
GLY
PHO
DOM
PEP
JNC
EXC
EF−hand
EH 1
EH 2
SH3 1
SH3 2
SH3 3
SH3 4
SH3 5
DH
PH
C2
Predicted Sequence Disorder
1.0
0.5
0.0
0
500
1000
1500
POS
Ds
0.6
0.4
0.2
modType
Phosphoserine
Phosphothreonine
Phosphotyrosine
B
Distribution of sequence disorder in excised
vs. mapped and non−excised regions of protein
M−W P−value vs. mapped: 9.88e−15 vs. non−excised: 3.36e−13
Predicted Sequence Disorder
1.00
0.75
0.50
0.25
0.00
ExcisedRegion
Mapped
NonExcisedRegion
C
Enrichment of phosphosites in skipped exons
spanned by identified splice junction
Fisher's exact test P: 1
Rest of Protein
Excised Region
9
255
0
3
pS/T/Y
S/T/Y
